# Supplementary material for: Subjective burden of government-imposed Covid-19 restrictions in Switzerland: Evidence from the 2022 LINK Covid-19 survey
Source: PLoS One. 2023 Jul 27;18(7):e0283524. doi: 10.1371/journal.pone.0283524 (PMC10374048; doi:10.1371/journal.pone.0283524)
Supplement: S1 Appendix — (DOCX) [file pone.0283524.s001.docx]

**Appendix 1: Original Questions in French, German and Italian**

***French version***

"Bienvenue à l'enquête en ligne sur le coronavirus (Covid-19) de l'Institut LINK. Merci beaucoup de participer à l'enquête.

En tant que principal institut d'études de marché et de recherche sociale en Suisse, nous souhaitons utiliser cette enquête pour montrer comment les suisses évaluent le thème du coronavirus et comment il influence leur vie quotidienne.

L'enquête ne dure que 10 minutes environ et fournit des informations importantes sur l'acceptation des mesures officielles et l'état d'esprit général de la population."

"Enfin, nous aimerions savoir dans quelle mesure les restrictions imposées par les gouvernements peuvent affecter notre qualité de vie personnelle.

Supposez un monde sans COVID-19. Vous devez prendre une décision entre vivre dans deux pays, avec des salaires différents ainsi que des restrictions différentes sur la vie quotidienne. Dans lequel des deux pays préféreriez-vous vivre et travailler ?

Pays A

Pays B

Je voudrais maintenant vous interroger sur des scénarios spécifiques. Dans chacun de ces scénarios, je voudrais que vous réfléchissiez à votre propre vie telle que vous la vivez habituellement, puis que vous la compariez à une vie alternative avec des restrictions. Supposez que toutes les autres choses soient les mêmes, c'est-à-dire ne considérez que votre situation privée.

Tout d'abord, veuillez envisager un scénario de restrictions en cas de pandémie de Covid-19, où vous devez porter un masque à tout moment en public, vous n'êtes pas autorisé à sortir pour dîner, boire un verre, aller en boîte ou à la salle de sport, et les voyages sont interdits. Si vous deviez faire un choix entre la vie avec ces restrictions et votre vie habituelle :

..préféreriez-vous avoir <X> mois de votre vie habituelle (option A) ou 12 mois dans ce type d'enfermement strict (option B) ?

Option A: vie normale

Option B: vie restreinte

Indifférent

Maintenant, veuillez envisager à la place un scénario de verrouillage encore plus strict, où vous devez porter des masques dans tous les espaces publics, vous ne pouvez pas sortir pour dîner, boire un verre, aller dans des clubs ou à la salle de sport, les fêtes et événements privés sont interdits, tous les enfants doivent recevoir un enseignement à distance tout en restant à la maison, et vous n'êtes pas autorisé à voyager. Si vous deviez faire un choix entre la vie dans ce type de verrouillage et votre vie habituelle :

..préféreriez-vous avoir X mois de votre vie habituelle (option A) ou 12 mois dans ce type d'enfermement strict (option B) ?

Option A: vie normale

Option B: vie restreinte

Indifférent

***German Version***

"Herzlich willkommen zur Online-Befragung zum Thema Coronavirus (Covid-19) des LINK Instituts. Besten Dank, dass Sie an der Befragung teilnehmen.

Als führendes Markt- und Sozialforschungsinstitut der Schweiz wollen wir durch diese Befragung aufzeigen, wie die Schweizerinnen und Schweizer das Thema Coronavirus einschätzen und wie es ihren Alltag beeinflusst.

Die Befragung dauert nur ca. 10 Minuten und liefert wichtige Erkenntnisse über die Akzeptanz der behördlichen Massnahmen und die allgemeine Stimmung in der Bevölkerung."

Stellen Sie sich eine Welt ohne COVID-19 vor. Sie müssen sich für ein Leben in einem der folgenden zwei Länder entscheiden. Die Länder unterscheiden sich sowohl im Gehalt das Sie verdienen, als auch in den Restriktionen, welche die Regierung für den Alltag beschlossen hat. In welchem der beiden Länder würden Sie vorziehen zu leben und zu arbeiten?

Land A

Land B

Ich möchte Sie nun zu bestimmten Szenarien befragen. In jedem dieser Szenarien möchte ich Sie bitten, über Ihr eigenes Leben nachzudenken, wie Sie es normalerweise leben, und es dann mit einem alternativen Leben mit Einschränkungen zu vergleichen. Gehen Sie davon aus, dass alle anderen Dinge gleich sind, d.h. betrachten Sie nur Ihre private Situation.

Betrachten Sie zunächst ein Szenario, bei dem Sie in der Öffentlichkeit immer eine Maske tragen müssen, nicht in Restaurants, in Clubs oder ins Fitnessstudio gehen dürfen und Reisen verboten sind. Wenn Sie die Wahl hätten zwischen einem Leben mit diesen Einschränkungen und Ihrem normalen Leben:

...würden Sie lieber <X> Monate lang Ihr normales Leben führen (Option A) oder 12 Monate lang in dieser Art von strengem Lockdown (Option B)?

Option A: normales Leben

Option B: eingeschränktes Leben

Gleichgültig

Stellen Sie sich stattdessen ein noch strengeres Abriegelungsszenario vor, in dem Sie in allen öffentlichen Räumen Masken tragen müssen, nicht zum Essen, Trinken, in Clubs oder ins Fitnessstudio gehen können, private Partys und Veranstaltungen verboten sind, alle Kinder zu Hause unterrichtet werden müssen und Sie nicht reisen dürfen. Wenn Sie die Wahl hätten zwischen einem Leben in dieser Art von Abriegelung und Ihrem normalen Leben:

...würden Sie lieber <X> Monate lang Ihr normales Leben führen (Option A) oder 12 Monate lang in dieser Art von strengem Lockdown (Option B)?

Option A: normales Leben

Option B: eingeschränktes Leben

Gleichgültig

***Italian Version***

Benvenuti al sondaggio online sul tema Coronavirus (Covid-19) dell'Istituto LINK. La ringraziamo in anticipo per la partecipazione al sondaggio.

In qualità di istituto di ricerca sociale e di mercato leader in Svizzera, attraverso questo sondaggio vorremmo mostrare come la popolazione svizzera valuta il tema Coronavirus e come esso influenza sulla sua vita quotidiana.

Il sondaggio dura solo 10 minuti circa e fornisce importanti informazioni sull'accettazione delle misure ufficiali e sullo stato d'animo generale della popolazione.

Infine, vorremmo sapere quanto le restrizioni imposte dai governi abbiano influenzato la qualità della vita delle persone.

Immagini di vivere in un mondo senza COVID-19. Deve scegliare tra vivere in due paesi, con stipendi diversi e diverse restrizioni alla vita quotidiana. In quale dei due paesi preferebbe vivere e lavorare?

Paese A

Paese B

Ora vorrei chiederti di immaginarti degli scenari specifici. In ognuno di questi scenari, vorrei che pensassi alla tua vita come la vivi di solito, e poi la confrontassi con una vita alternativa con restrizioni. Assumi che tutte le altre cose siano rimaste le stesse, cioè considera solo la tua situazione privata.

Per prima cosa, considera uno scenario di restrizioni leggere per contenere la pandemia, dove all’esterno e nei luoghi pubblici (all’interno?) è obbligatorio indossare sempre una maschera facciale; non è consentito andare a ristoranti, bar, discoteche e palestre, e non è consentito viaggiare per turismo. Se dovessi fare una scelta tra la vita con queste restrizioni e la tua vita abituale:

..preferiresti avere <X> dei mesi di vita senza restrizioni (opzione A) o 12 dei mesi di vita con queste particolare restrizioni (Opzione B)?

Opzione A: vita normale

Opzione B: vita limitata

Indifférente

Ora supponi che nel tuo paese vi siano ulteriori restrizioni, nello specifico, nel tuo paese è obbligatorio indossare le maschere facciali in tutti i luoghi pubblici, non è consentito andare in ristoranti, bar, discoteche e palestre; le feste e gli eventi privati sono vietati, non è possibile frequentare asili, scuole materne, scuole elementari, medie inferiori e superiori, esiste solo l’insegnamento a distanza; e non è consentito viaggiare per turismo. Se dovessi fare una scelta tra la vita con questo tipo di ulteriori restrizioni e la tua vita abituale:

..preferiresti avere <X> dei mesi di vita senza restrizioni (opzione A) o 12 dei mesi di vita con queste particolare restrizioni (Opzione B)?

Opzione A: vita normale

Opzione B: vita limitata

Indifferente
