# Supplementary material for: Subjective burden of government-imposed Covid-19 restrictions in Switzerland: Evidence from the 2022 LINK Covid-19 survey
Source: PLoS One. 2023 Jul 27;18(7):e0283524. doi: 10.1371/journal.pone.0283524 (PMC10374048; doi:10.1371/journal.pone.0283524)
Supplement: S2 Appendix — (DOCX) [file pone.0283524.s002.docx]

**Appendix 2: DCE Examples**

**Vignette 1**


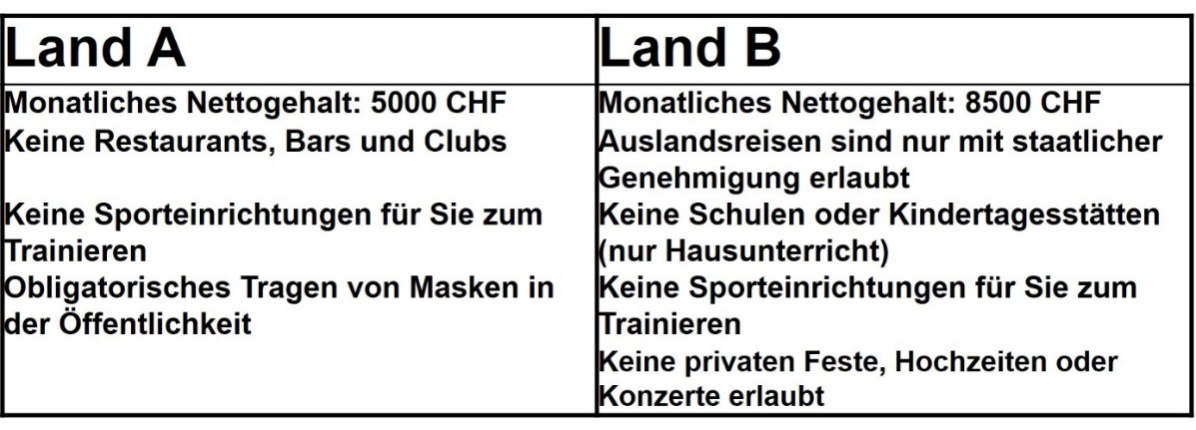


Decision pattern observed: 64% choose option A, 36% option B.

Translation:

**Vignette 3**


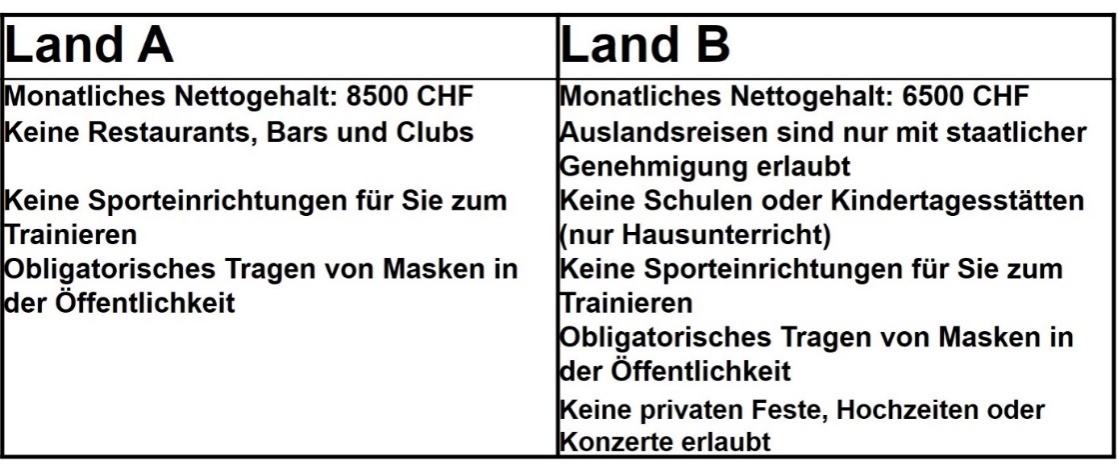


Decision pattern observed: 91.7%% choose option A, 8.3 % option B.

**Translation**

**Vignette 24**


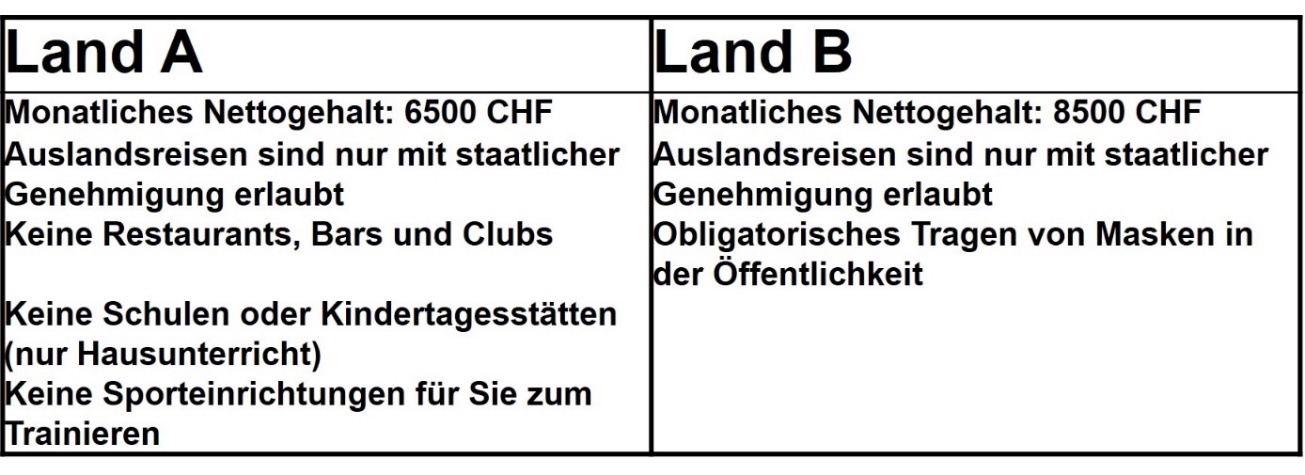


Decision pattern observed: 11.7%% choose option A, 88.3 % option B.

**Translation**
