# Supplementary material for: Subjective burden of government-imposed Covid-19 restrictions in Switzerland: Evidence from the 2022 LINK Covid-19 survey
Source: PLoS One. 2023 Jul 27;18(7):e0283524. doi: 10.1371/journal.pone.0283524 (PMC10374048; doi:10.1371/journal.pone.0283524)
Supplement: S1 Fig — (DOCX) [file pone.0283524.s003.docx]

**Appendix Figure AF1: Oxford Stringency Index Switzerland**
