# Supplementary material for: Subjective burden of government-imposed Covid-19 restrictions in Switzerland: Evidence from the 2022 LINK Covid-19 survey
Source: PLoS One. 2023 Jul 27;18(7):e0283524. doi: 10.1371/journal.pone.0283524 (PMC10374048; doi:10.1371/journal.pone.0283524)
Supplement: S2 Fig — (DOCX) [file pone.0283524.s004.docx]

**Appendix Figure AF2: Time-tradeoffs light restrictions**

Based on the question “*Imagine a scenario where you are required to wear masks in all public spaces, cannot go out to eat or have drink, you cannot go to clubs or the gym, and you are not allowed to travel. Would you rather have x months of your normal life, or 12 months of life with these restrictions?*” Frequencies represent unweighted counts.
