# Supplementary material for: Subjective burden of government-imposed Covid-19 restrictions in Switzerland: Evidence from the 2022 LINK Covid-19 survey
Source: PLoS One. 2023 Jul 27;18(7):e0283524. doi: 10.1371/journal.pone.0283524 (PMC10374048; doi:10.1371/journal.pone.0283524)
Supplement: S1 Table — (DOCX) [file pone.0283524.s005.docx]

**Appendix Table AT1: Modeling details for QALY calculations**

| **Restrictions imposed between January 1, 2020 and February 28, 2022** | | |
| --- | --- | --- |
|  |  |  |
| **Level of restrictions** | **Days** |  |
|  |  |  |
| Strict: > 70 | 41 |  |
| Medium: 50-70 | 392 |  |
| Light: 20-49 | 295 |  |
|  |  |  |
| *Source* |  |  |
| Oxford Covid-19 Government Response Tracker (https://doi.org/10.1038/s41562-021-01079-8) | | |
|  |  |  |
|  | | |
| **Scenario 1** | **Utility weight** | **Source** |
|  |  |  |
| Severe restrictions | 0.392 | Severe scenario, excluding extreme preferences |
| Moderate restrictions | 0.595 | Interpolation |
| Light restrictions | 0.797 | Interpolation |
|  |  |  |
| **Scenario 2** |  |  |
|  |  |  |
| Severe restrictions | 0.298 | Severe scenario, all responses |
| Moderate restrictions | 0.325 | Mild scenario, all responses |
| Light restrictions | 0.676 | Interpolation |
|  |  |  |
|  |  |  |
| **Scenario 3** |  |  |
|  |  |  |
| Severe restrictions | 0.593 | Severe scenario, excluding extreme preferences |
| Moderate restrictions | 0.729 | Interpolation |
| Light restrictions | 0.864 | Interpolation |
